# Disruption of BDNF signalling in neuropathologies

**DOI:** 10.1042/BST20253079

**Published:** 2026-02-23

**Authors:** Aurélie Paulo-Ramos, Elena R. Rhymes, David Villarroel-Campos, James N. Sleigh

**Affiliations:** 1 Department of Neuromuscular Diseases and UCL Queen Square Motor Neuron Disease Centre, UCL Queen Square Institute of Neurology, University College London, London, U.K.; 2 UK Dementia Research Institute, University College London, London, U.K.

**Keywords:** amyotrophic lateral sclerosis (ALS), brain-derived neurotrophic factor (BDNF), neurotrophin, tropomyosin receptor kinase B (TrkB)

## Abstract

The vital role of brain-derived neurotrophic factor (BDNF) in neuronal development, synaptic plasticity, and neuroprotection has been explored for decades. Therefore, the expression, processing, and signalling activities of this neurotrophin, which is reliant upon TrkB and p75^NTR^ receptors, have been well characterised in both health and disease. This review summarises the latest findings on BDNF dysregulation in neuropathologies. Indeed, across diseases of both the central and peripheral nervous systems, BDNF signalling is frequently disrupted, contributing to neuronal dysfunction and degeneration. Consequently, through direct or indirect enhancement of its expression and/or function, BDNF has proved to be a promising therapeutic target across many neurological conditions. However, the complexity of its regulation and interaction with several different receptors underpins the need for further research to deepen our understanding of BDNF disruption in neuropathologies and to achieve its therapeutic potential.

## Introduction

Brain-derived neurotrophic factor (BDNF) is a member of the neurotrophin family of growth factors, which also includes nerve growth factor, neurotrophin-3, and neurotrophin-4/5. Widely expressed across neural tissue, BDNF is a critical neurotrophic factor in both the central (CNS) and peripheral nervous systems (PNS), where it promotes neuronal differentiation, growth, and survival through binding to its high-affinity receptor, tropomyosin receptor kinase B (TrkB). This receptor–ligand interaction initiates pro-survival signalling, which is dependent on activation of three key downstream pathways: PI3K/AKT, MAPK/ERK, and PLC [1]. BDNF-TrkB signalling can subsequently engage the transcription factor ‘cAMP response element-binding protein’ (CREB), along with its co-activator ‘CREB-binding protein’, to drive gene transcription that modulates neurotransmission and supports neuronal plasticity (e.g., long-term potentiation) [2].

Accordingly, BDNF/TrkB impairments have been implicated in the pathophysiology of many neurological disorders, ranging from psychiatric to neurodegenerative [3]. Indeed, decreased BDNF function, whether through reduced mRNA or protein levels, impaired synthesis/secretion, or disrupted signalling, has been associated with major neuropathologies, such as Alzheimer’s disease (AD), Huntington’s disease (HD), and Parkinson’s disease (PD) [4].

BDNF is also expressed in peripheral nerves and tissues, including endothelial and glial cells, as well as smooth and skeletal muscles [3]. Moreover, BDNF has been shown to play clear roles in sensory neuron and neuromuscular development, including in the formation and maturation of neuromuscular junctions (NMJs), as well as muscle fibre type specification. Accordingly, impaired BDNF signalling has also been identified in diseases impacting the PNS, such as amyotrophic lateral sclerosis (ALS), spinal muscular atrophy (SMA) [5], and Charcot–Marie–Tooth disease (CMT) [6].

**Figure 1:**
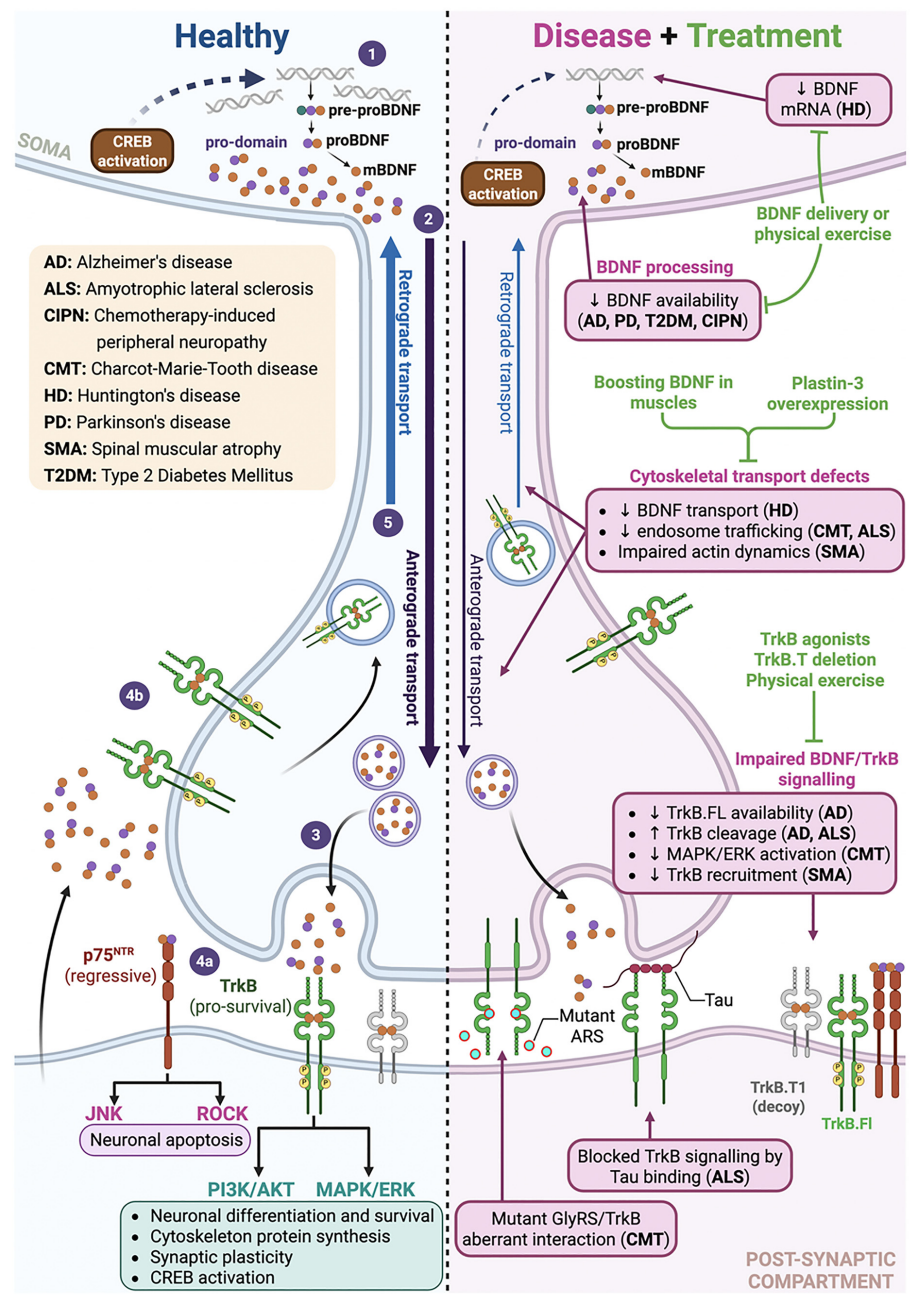
Summary of BDNF processing and signalling in healthy versus neuropathological neurons with potential treatment strategies In healthy neurons (left side, blue), BDNF processing results in the production of both proBDNF and mature BDNF (mBDNF) (**1**). These forms undergo anterograde axonal transport (**2**) and are secreted at the synaptic cleft (**3**). Secreted proBDNF binds to the p75 neurotrophin receptor (p75^NTR^), activating regressive signalling pathways, while mBDNF binds to post-synaptic tropomyosin receptor kinase B (TrkB) receptors, initiating pro-survival signalling (**4a**). Alternatively, mBDNF can come from the post-synaptic compartment (e.g., muscle) and bind to pre-synaptic TrkB receptors (**4b**); the resulting activated complex undergoes retrograde axonal transport, promoting the activity of the transcription factor CREB in the nucleus, which is essential for further BDNF production (**5**). Disruption of BDNF processing and signalling is observed in various neuropathological conditions (right side, pink); accordingly, multiple therapeutic approaches aim to mitigate the deleterious effects associated with these disruptions (green).

Recent comprehensive reviews have examined the role of BDNF across neurodegenerative diseases [7–10], but peripheral neurodegeneration is often not discussed to the same extent. This review bridges this gap by identifying convergent pathological mechanisms throughout the nervous system while critically evaluating the translational challenges of harnessing BDNF that have restricted clinical success in spite of the considerable preclinical promise (Figure 1).

## Biology of BDNF

### BDNF synthesis and expression

The gene encoding BDNF has 11 exons in humans and 9 in rodents and is initially translated into the precursor protein pre-proBDNF, which undergoes processing in the endoplasmic reticulum (ER) to produce proBDNF. After trafficking to the Golgi apparatus and reaching the *trans*-Golgi network, proBDNF is sorted into vesicles for secretion, after which it undergoes intracellular or extracellular cleavage of the N-terminal pro-domain, creating mature BDNF (mBDNF).

BDNF isoform levels are developmentally regulated. Indeed, it has been shown that proBDNF is highly expressed in mouse brain in the early postnatal period. However, a shift is observed as the nervous system matures, with mBDNF becoming predominant from adolescence and throughout adulthood [11]. The conversion of proBDNF to mBDNF is associated with increased expression and activity of extracellular proteases, including matrix metalloproteinases [12]. Regional variations in this regulation have been identified, such as the increased mBDNF in cortical and hippocampal structures during synaptogenesis and circuit refinement, and the relative abundance of proBDNF in regions undergoing active remodelling or in pathological states where proteolytic processing is impaired [13–15].

BDNF is expressed broadly, and its release is modulated by context-specific stimuli. For example, proBDNF and mBDNF secretion is tightly regulated by neuronal activity and Ca^2+^-dependent signalling pathways. During synaptic maturation, electrical activity patterns trigger BDNF expression, which varies depending on the developmental stage [16]. While in non-electrically excitable cells, such as glial cells, BDNF secretion is primarily regulated by the binding of extracellular nucleotides, neuropeptides, and pro-inflammatory factors to their respective receptors [17]. A notable single nucleotide polymorphism in the *BDNF* gene, rs6265, which generates the Val66Met substitution, alters synaptic targeting of BDNF and impairs its activity-dependent release. Val66Met has been associated with increased susceptibility to several neurodegenerative diseases, underscoring the critical role of activity-dependent BDNF secretion in maintaining nervous system function [18].

### BDNF receptors and signalling

BDNF signalling is mediated by two main receptors; mBDNF primarily binds to TrkB, a high-affinity receptor tyrosine kinase, but can also bind to the low-affinity p75 neurotrophin receptor (p75^NTR^), a member of the tumour necrosis factor receptor family [19].

mBDNF binding to TrkB causes receptor dimerisation and cross-phosphorylation of key intracellular tyrosine residues, leading to activation of progressive signalling pathways. This can occur locally at the plasma membrane or distally, via internalisation of activated BDNF-TrkB complexes into signalling endosomes, for long-range delivery to neural somas via the process of retrograde axonal transport [20]. The two most extensively studied pro-survival pathways are PI3K/AKT and MAPK/ERK. PI3K/AKT signalling is crucial for promoting neuronal survival and differentiation, as well as for supporting synaptic plasticity and dendritic growth. MAPK/ERK signalling regulates protein synthesis during neuronal development and plays a pivotal role in CREB activation, which facilitates transcription of genes involved in synaptic potentiation, dendritic arborisation, and neuronal differentiation and survival [21].

In contrast, proBDNF binding to p75^NTR^ and sortilin receptors [22] induces broadly regressive pathways [23]. For instance, formation of the ligand-receptor complex activates the ‘c-Jun amino-terminal kinase’ pathway that leads to neuronal apoptosis, caspase activation, and dendritic spine loss, while increased ‘RhoA/Rho-associated kinase’ signalling inhibits PI3K/AKT, also contributing to apoptosis. ProBDNF thus acts in opposition to mBDNF, making the proteolytic cleavage of BDNF a balancing mechanism between progressive and regressive neuronal events [24].

## Disruption of BDNF signalling in neuropathologies

### Alzheimer’s disease

AD predominantly affects the elderly, with a prevalence of 10%–30% in individuals >65 yr old. AD is characterised by toxic accumulation in the brain of amyloid beta peptide (Aβ), as well as the microtubule‐associated protein tau, which aggregates into neurofibrillary tangles; this leads to progressive decline in cognitive function, particularly memory, along with neuronal loss and synaptic dysfunction. In addition to processing amyloid precursor protein into Aβ, δ-secretase cleaves both tau and TrkB to create a self-amplifying cascade of neurodegeneration. This leads to the generation of truncated tau fragments that aggregate and exhibit neurotoxicity [25,26]. Critically, these fragments directly bind to TrkB, blocking neurotrophic signalling even in the presence of BDNF [27]. Simultaneously, δ-secretase-mediated TrkB cleavage generates a truncated receptor with impaired signalling properties. Intact TrkB normally acts to protect against Aβ production, but its cleavage abolishes this function [26], resulting in increased Aβ production and accumulation, which in turn further activates δ-secretase. More tau fragments are produced, inhibiting functional TrkB receptors and simultaneously generating more dysfunctional TrkB fragments. This drives tau pathology, which further diminishes neurotrophic signalling, leading to neurodegeneration [27,28].

Given its importance for neuronal function, synaptic plasticity, learning and memory, BDNF has emerged as a key protein of interest in AD. Indeed, in a meta-analysis of expression in AD patient brains, *BDNF* was identified as a critical AD gene, with expression clearly declining with disease progression and negatively correlating with Aβ levels [29]. Reduced availability of BDNF has also been observed in the hippocampus and prefrontal cortex of a streptozotocin-induced AD mouse model, associated with cognitive decline and neurodegeneration [30]. Moreover, lower expression of BDNF and TrkB coincides with characteristic features of AD, such as neuritic plaques and neurofibrillary tangles, in human pyramidal and hippocampal neurons [31]. Similarly, BDNF-mediated TrkB trafficking and activation of downstream MAPK/ERK pro-survival signalling are perturbed in mouse basal forebrain neurons from an AD–Down syndrome model [32]. Additionally, experimental BDNF deprivation induces activation of δ-secretase in mouse cortical neurons, resulting in proteolytic cleavage of TrkB and tau, thereby further attenuating neurotrophic signalling and exacerbating neurodegenerative cascades [27]. Consistent with this mechanism, increased levels of cleaved TrkB and reduced levels of TrkB.FL are observed in patient cerebrospinal fluid (CSF) as AD progresses, reflecting pathological receptor processing [33]. Accordingly, genetic or pharmacological inhibition of δ-secretase-mediated cleavage of tau or TrkB improves amyloid pathology, cognitive defects and synaptic dysfunction in the most studied AD mouse model, 5xFAD mice, supporting a causal role for impaired BDNF-TrkB signalling in disease progression [26,27,33]. Moreover, altered levels of BDNF in the hippocampus have been linked to an elevation of phosphorylated tau levels in post-mortem AD brains, potentially influencing amyloid accumulation [34]. In addition to neuronal and synaptic damage, myelin deficits can also occur in AD. A study in 5xFAD mice highlighted that recombinant BDNF can protect oligodendrocyte progenitor cells from apoptosis *in vitro* while promoting their proliferation and differentiation, suggesting that disrupted BDNF signalling may contribute to both neuronal and myelin pathology [35]. These findings underscore the neuroprotective role of BDNF in glial cell function, suggesting that enhancing BDNF signalling may help to mitigate AD-related neurodegeneration.

The evidence for the use of BDNF as a biomarker in AD is conflicting; both decreased [36–39] and increased [40–45] BDNF levels have been identified in the plasma/serum of patients compared with controls, a discrepancy indicating that circulating BDNF is not a reliable indicator of AD.

Nevertheless, BDNF remains a key factor in AD development, leading to efforts to enhance its function. For instance, the effects of boosting BDNF signalling in AD models have been studied *in vivo* and *in vitro* via injection or transplantation of overexpressing cells, lipid-based nanoparticle transfer, provision of TrkB agonist compounds, and intranasal delivery of a recombinant BDNF solution [28,46–55]. These approaches ameliorated AD pathology, causing reductions in inflammation, Aβ production, plaque deposition, and neuronal apoptosis, and were associated with improved cognitive function, neurogenesis, and synaptic plasticity. Furthermore, expression of a signalling‐deficient variant of p75^NTR^ in 5xFAD mice is neuroprotective [56], while p75^NTR^ modulation by small molecule LM11A-31 was recently shown in a Phase IIa trial to slow progression of AD pathophysiology [57]. Collectively, these studies indicate that targeting BDNF-TrkB and its related pathways could be beneficial during AD pathogenesis. Accordingly, a Phase I clinical trial is currently recruiting a small number of AD and mild cognitive impairment patients to assess safety, tolerability, and preliminary efficacy of an adeno-associated virus (AAV) gene therapy designed to increase BDNF in the brain [58]—results are expected in late 2027.

### Huntington’s disease

HD is caused by a CAG trinucleotide repeat expansion in the first exon of the huntingtin gene, *HTT*. Clinically, HD manifests with motor and cognitive impairments and a range of emotional and psychiatric disturbances resulting from degeneration of the medium spiny neurons in the striatum, which play a role in the regulation of voluntary and involuntary movements.

Similar to AD, the usefulness of BDNF as a biomarker in HD is unclear. Indeed, one study showed no difference in BDNF levels in CSF and plasma from HD patients [59], whereas another report found decreased BDNF levels in saliva [60]. Nevertheless, down-regulation of BDNF mRNA has been observed in post-mortem HD prefrontal cortex [61].

The disruption of anterograde axonal transport of BDNF from cortical neurons to the striatum caused by mutant huntingtin (mHTT) plays an important role in this pathogenesis. BDNF is usually synthesised predominantly in cortical pyramidal neurons and actively transported along corticostriatal axons to the striatum, where it provides essential trophic support to medium spiny neurons expressing high levels of TrkB receptors [62]. This process requires the formation of a complex involving wild-type huntingtin, huntingtin-associated protein-1 (HAP1), dynactin, and kinesin-1 to promote BDNF vesicular transport along microtubules [63,64]. However, mHTT binds HAP1 and dynactin with a higher affinity than wild-type HTT, disrupting their association with microtubules, effectively slowing down BDNF-containing vesicles [65,66]. The consequence is a reduction in BDNF delivery and activity-dependent secretion in the HD striatum, despite relatively preserved cortical synthesis, leading to selective trophic deprivation of striatal neurons [62]. Notably, striatal neurons show enhanced sensitivity to these transport defects compared with cortical or hippocampal neurons [65], and both anterograde transport of BDNF and activity-dependent BDNF secretion in the striatum are severely disrupted in HD mouse models [65,67]. Recently, primary cortical and striatal neurons isolated from an HD mouse model with 140 CAG repeats (Q140 mice) showed reduced anterograde and retrograde transport of BDNF from cortical-to-striatal and striatal-to-cortical neurons, respectively, evidenced as reduced movements and prolonged pauses without a decline in moving speed [68]. This impaired BDNF transport limits neurotrophic support, which could exacerbate striatal neuron degeneration, positioning BDNF trafficking, rather than global BDNF deficiency, as central mechanistic driver of HD pathology, supporting therapeutic strategies aimed at restoring BDNF delivery or signalling.

Indeed, the direct enhancement of BDNF availability via systemic, chronic administration of recombinant BDNF restored hippocampal viability and CREB phosphorylation in an HD mouse model [69]. Additionally, the transplantation of BDNF-overexpressing human neural stem cells into the striatum of a rat model, with a chemically-induced striatum lesion, resulted in behavioural improvement and reduced inflammatory response [70]. Similarly, the intrastriatal transplantation of striatal progenitor cells, engineered to overexpress BDNF, caused a reduction in mHTT aggregates and improved cognitive and motor functions [71]. Treatment of HD mouse primary cortical neurons with BDNF also rescued synaptic and functional deficits [72]. Finally, combined delivery via intranasal and intraperitoneal routes of LM22B-10, a small molecule ligand that activates both TrkB and TrkC, caused increased TrkB/C phosphorylation, enhanced AKT signalling, and improved motor performance in two different HD mouse models [73]. Taken together, these results highlight enhancing BDNF and TrkB signalling as a potential therapeutic strategy for HD.

### Parkinson’s disease

PD is characterised by the progressive loss of dopaminergic neurons within the substantia nigra, leading to tremor, rigidity, bradykinesia, and postural instability along with psychiatric symptoms. This can be associated with the aggregation of α-synuclein within neurons, leading to degeneration. Decreased levels of BDNF in the brain and plasma have been reported in PD patients [74–78] and may contribute to the development of depressive symptoms [79,80]. Several studies indicate that the BDNF Val66Met polymorphism may exacerbate PD, emphasising the importance of BDNF secretion [81–85]. Recently it was shown that dopamine depletion in cultured direct pathway striatal medium spiny neurons, a rat PD model, and postmortem PD brains causes TrkB accumulation and clustering within the ER, which restricts lysosomal degradation, thus impairing TrkB processing [86]. Moreover, BDNF reduction in the gut–brain axis also appears to be associated with PD development [87,88]. It is suggested that dysbiosis in the gastrointestinal system promotes intestinal permeability, gastrointestinal inflammation, and α-synuclein aggregation [89]. Environmental factors, including the gastrointestinal microbiota, induce a progressive accumulation of α-synuclein within the extracellular matrix, leading to oxidative stress and mucosal inflammation [90]. This may promote the misfolding and aggregation of endogenous α-synuclein in enteric neurons, which is then disseminated through the enteric and the autonomic nervous system to the CNS [91,92]. In mice, experimental gut injection of α-synuclein fibrils that convert endogenous α-synuclein to a pathologic form, capable of spreading to the brain, leads to motor and non-motor features of PD [93]. This pathological α-synuclein could disseminate retrogradely via the vagal nerve, which represents a direct anatomical connection between the gut and the brainstem, triggering dopaminergic neurodegeneration [91]. Supporting this model, full truncal vagotomy is associated with a decreased risk for subsequent PD, suggesting that the vagal nerve may be critically involved in the pathogenesis of PD [94]. Other studies have shown mixed results however, with some suggesting that protective effects only emerge after extended follow-up periods [95]. Experimentally, vagotomy and α-synuclein deficiency prevent the neurodegeneration and neurobehavioural deficits induced by gut-transmitted α-synuclein [93]. Interestingly, gut dysbiosis is associated with reduced circulating BDNF levels, potentially reflecting diminished microbial production of BDNF-inducing metabolites, but conversely, BDNF may modulate the gut barrier integrity and inflammation, suggesting that BDNF deficiency could both result from and contribute to the gut-brain pathological cascade in PD [96,97].

Stimulation of BDNF expression and signalling has consequently gained attention as a potential therapeutic strategy for PD. Accordingly, BDNF overexpression has neuroprotective effects in rodent PD models, promoting autophagy, inhibiting neuronal apoptosis, lowering motor and cognitive deficits, and improving working memory, which altogether slow disease progression [98–100]. Additionally, administration of CFC3N, an optimised 7,8-dihydroxyflavone (7,8-DHF) TrkB agonist, has shown promising results, activating neurotrophic pathways in PD mouse models and promoting dopaminergic neuron survival [101]. Furthermore, use of 7,8-DHF on rat and mouse PD models activated BDNF-TrkB signalling and rescued dopaminergic neuron loss, motor function, and mitochondrial alterations [102–104]. Finally, in line with findings in other neurodegenerative disorders, physical exercise has been shown to increase serum BDNF levels in PD patients and correlate with alleviation of motor symptoms [105], with the general caveat that the positive effects of exercise may not be limited to BDNF. A Phase III randomised clinical trial is currently underway to assess the impact of exercise on PD progression, which may further support the role of BDNF in disease management [106].

### Amyotrophic lateral sclerosis

ALS is characterised by the progressive loss of upper and lower motor neurons (MNs), causing severe muscle atrophy, paralysis, and impairment of essential functions, such as swallowing and breathing. The aetiology of ALS is multi-factorial, with protein misfolding, oxidative stress, abnormal cell signalling, altered RNA processing, defective axonal transport, and a deficiency in neurotrophic support all contributing to disease. BDNF-TrkB signalling is dysregulated in ALS, leading to its extensive study as a therapeutic target [5]. Specifically, studies found a distinctive role of the truncated form of TrkB, TrkB.T1, which is known to compete with TrkB.FL for BDNF binding without triggering the classical pathways [107]. Indeed, a widely studied ALS model, the superoxide dismutase type 1 mutant mouse (SOD1^G93A^), was shown to display a selective reduction in the speed of neurotrophic signalling endosome axonal transport specifically in fast lower MNs [108]. In addition, an impaired response to neurotrophins was also detected, characterised by an absence of rescued endosome trafficking upon BDNF augmentation in muscles of ALS compared with wild-type mice. A similar absence of effect was shown in primary MNs derived from SOD1^G93A^ mice. Perhaps accounting for this refractory phenotype, levels of p75^NTR^ and TrkB.T1 were found to be increased in muscles, sciatic nerves, and Schwann cells of SOD1^G93A^ mice, suggesting that dysregulation of these receptors may contribute to ALS pathogenesis by disrupting effective TrkB.FL signalling [108]. Supporting this hypothesis, deletion of TrkB.T1 delays the phenotypic onset in SOD1^G93A^ mice in a non-cell autonomous manner [109].

Like in PD, physical exercise exerts beneficial effects on BDNF-TrkB signalling in ALS mice [110]. Exercise was found to restore the balance between mBDNF and proBDNF in muscles of the SOD1^G93A^ model. This ratio of BDNF isoforms is also affected in ALS patient CSF, where a marked increase in mBDNF and decrease in proBDNF are associated with enhanced disease progression and shorter survival, probably related to a change in the TrkB.T1 to TrkB.FL ratio [111]. Furthermore, swimming restored both of these ratios, which in turn mitigated neurotrophic signalling alterations and prevented MN loss in SOD1^G93A^ mice [110]. These results demonstrate the preventative approach of exercise in ALS to promote neurotrophic signalling, preserve neuromuscular function, and delay disease progression. Corroborating this, a 7,8-DHF pro-drug called R13, which selectively activates TrkB signalling, has been shown to enhance motor performance and reduce muscle atrophy in SOD1^G93A^ mice [112]. Interestingly, selective expression of BDNF, via muscle delivery of an AAV-mediated therapy, delayed disease onset and slowed progression in SOD1^G93A^ mice [113].

### Charcot–Marie–Tooth disease

CMT is a hereditary motor and sensory neuropathy characterised by progressive degeneration of peripheral nerves, resulting in muscle weakness and sensory defects, mainly in distal limbs. The condition is highly genetically heterogeneous (>100 different associated genes) and divided into two principal subtypes based on the primary pathological target: CMT1 (demyelinating) and CMT2 (axonal). Many of the genes associated with CMT encode proteins involved in essential cellular processes, such as axonal transport, mitochondrial function and endosomal sorting.

While BDNF has been largely unexplored in CMT pathogenesis, our recent studies provide insights into its potential involvement. CMT2D mouse models harbouring dominantly inherited point mutations in *GARS1*, a member of the aminoacyl-tRNA synthetase (ARS) gene family that encodes glycyl-tRNA synthetase (GlyRS), display impairments in axonal transport of neurotrophic signalling endosomes that result from disruption in BDNF-TrkB signalling [6]. Mutant CMT2D-causing GlyRS aberrantly interacts with the extracellular domain of TrkB [114], and the availability of TrkB within muscles closely correlates with the extent of NMJ denervation in CMT2D mice, suggesting a role for this pathway in the selective vulnerability of MNs. Dampened MAPK/ERK signalling was observed in CMT2D nerves, while MAPK/ERK inhibition within wild-type muscles was sufficient to impair endosome axonal transport, indicating that BDNF at nerve terminals can non-cell autonomously regulate axonal endosome dynamics. Given these findings, BDNF expression was therapeutically targeted to CMT2D mouse skeletal muscles and shown to rescue axonal transport and MAPK/ERK signalling [6]. This approach also proved effective in combatting transport disruption in a mouse model for dominant intermediate CMT type C (DI-CMTC) caused by mutations in *YARS1* (encoding tyrosyl-tRNA synthetase), emphasising the relevance of muscle-targeted BDNF therapy in several CMT subtypes that result from ARS mutations [115].

Further investigations in CMT1 models revealed that physical exercise ameliorates certain pathophysiological features. In a CMT1X mouse model, exercise led to reduced inflammation, improved nerve architecture, and an increase in myelin thickness, which was associated with up-regulated BDNF expression in peripheral nerves [116]. These findings suggest that physical activity may augment BDNF signalling in neuropathy, potentially offering a complementary approach to pharmacological therapies in CMT1.

### Acquired peripheral neuropathy

In diabetic peripheral neuropathy (DPN), elevated serum BDNF levels have been reported to correlate with pain severity [117]. Supporting these observations, a rat model of DPN demonstrated increased BDNF expression in dorsal root ganglia and spinal cord, associated with the development of mechanical allodynia, which was attenuated by transiently inhibiting BDNF-TrkB signalling [118]. Conversely, other studies have highlighted the detrimental effects of BDNF reduction in acquired neuropathies. For example, type 2 diabetes patients displayed lower serum BDNF levels, associated with more severe neuropathy [119]. Reduced serum BDNF levels were also observed in patients with chemotherapy-induced neuropathy, suggesting that BDNF may be used as a biomarker for this particular neuropathy and that BDNF augmentation prior to treatment could be protective [120,121]. Indeed, BDNF supplementation prevented neuronal damage in a rat model of chemotherapy-induced neurotoxicity [122], while intravitreal injection of BDNF rescued visual function and prevented optic nerve degeneration in a porcine model of traumatic optic neuropathy [123]. Furthermore, DPN mice display reduced cutaneous BDNF expression and loss of TrkB-positive Meissner corpuscles, which can be rescued by restoring BDNF availability [124]. These findings indicate that, while excessive BDNF-TrkB signalling within sensory circuits can exacerbate neuropathic pain, insufficient BDNF supply in peripheral tissues accelerates nerve degeneration. Boosting BDNF thus appears to have a largely positive impact in the context of not only inherited but also acquired neuropathy, with the caveat of potentially elevating pain under certain circumstances.

### Spinal muscle atrophy and spinal-bulbar muscular atrophy

SMA is caused by loss-of-function in the *Survival of Motor Neuron 1* gene, resulting in lower MN degeneration, muscle weakness, and atrophy. Similar to ALS, primary MNs from SMA mice were shown to respond abnormally to BDNF, characterised by an impaired localisation of ribosomes and rough ER at motor axon terminals, resulting in a subsequent dampened local translation [125]. This defect is further compounded by impaired actin dynamics in SMA MNs, which limit TrkB recruitment to the plasma membrane *in vitro* and *in vivo*, leading to a decrease in BDNF-TrkB signalling at motor axon terminals [126]. Boosting levels of the actin-bundling protein plastin 3, which is a protective modifier of SMA, rescued actin dynamics and enhanced TrkB recruitment and activation at distal motor terminals, linking cytoskeletal dysregulation to deficient neurotrophic signalling in SMA pathophysiology [126].

Spinal-bulbar muscular atrophy (SBMA) is a progressive neuromuscular disease caused by CAG trinucleotide repeat expansions in the androgen receptor, predominantly affecting men and causing muscle weakness due to loss of lower MNs. Expression of *BDNF*, but not *TrkB*, was recently reported to be down-regulated both synaptically and extra-synaptically in muscles of two different SBMA mouse models [127]. Contrasting with this, *BDNF* mRNA expression was up-regulated in SBMA patient muscle and a third SBMA mouse model [128]. Nevertheless, genetic overexpression of BDNF in muscles of SBMA mice slowed disease progression through rescue of synaptic and muscle function [128]. Together, these studies indicate that impaired regulation and availability of BDNF-TrkB signalling at the neuromuscular interface is a shared mechanistic contributor to motor neuron vulnerability in SMA and SBMA, supporting translational potential for targeting BDNF-TrkB signalling.

### Other neuropathologies

BDNF plays a crucial role in endogenous repair and neuroplasticity following acute injury and is thus important in the context of traumatic brain injury (TBI). Unlike the progressive, cell-autonomous pathology characteristic of AD, PD, or HD, TBI is characterised by a biphasic response: firstly, involving excitotoxicity, oxidative stress, inflammation, and blood–brain barrier (BBB) disruption, followed by an endogenous repair phase notably characterised by neurogenesis, synaptogenesis, axonal sprouting, and circuit reorganisation. The role of BDNF in neural repair and regeneration following TBI is well established, with BDNF up-regulation occurring as an endogenous neuroprotective response [129]. This acute elevation of BDNF contrasts with the chronic deficiency observed in neurodegenerative diseases and drives TrkB-mediated survival signalling, enhances synaptic plasticity in spared circuits to compensate for lost function, and promotes neurogenesis. Regarding therapies, the temporal window of intervention appears crucial: voluntary exercise can endogenously up-regulate BDNF and enhance recovery after TBI, but when performed immediately post-injury, the molecular response to exercise is disrupted [130]. When interventions are given prior to damage, BDNF induction reduces neuronal degeneration and improves cognitive outcomes [131,132]. BDNF-related therapies have shown neuroprotection and increased neuroplasticity, counteracting brain injury-related deficits [133]. This context-dependent role of BDNF highlights that, in chronic degeneration, the therapeutic goal is to compensate for BDNF deficiency, whereas in TBI the goal is to amplify the endogenous BDNF-mediated repair response.

BDNF is also implicated in epileptogenesis [134], a common long-term complication of TBI, emphasising the dual role of BDNF and the need to balance its progressive and regressive effects. Post-traumatic epilepsy is one of the most devastating long-term network consequences of TBI and is characterised by enduring hyperexcitability and hypersynchrony [135]. This highlights a potential therapeutic challenge, since chronic BDNF elevation in the absence of appropriate activity-dependent regulation might promote maladaptive plasticity, including post-traumatic epileptogenesis and neuropathic pain hypersensitivity, underscoring the importance of temporal and spatial precision in BDNF-based interventions.

## Therapeutic strategies

### Limitations

Despite the promising evidence for BDNF-based interventions across multiple neuropathologies, several critical limitations have hampered clinical translation (Table 1). A fundamental challenge lies in the administration route for BDNF delivery. For instance, a large Phase III trial of recombinant BDNF in 1,135 ALS patients showed no significant survival benefit after nine months of treatment; however, BDNF was delivered subcutaneously, which is unlikely to effectively target the motor neurons impacted by the disease [136]. As detailed by Barde [7], the failure of early BDNF clinical trials such as this can be attributed to three fundamental obstacles. First, BDNF exhibits unfavourable pharmacokinetics, with rapid proteolytic degradation in serum and a half-life of only minutes following systemic administration [137]. Second, as a large, highly charged protein, BDNF cannot cross the BBB, preventing peripherally administered BDNF from reaching the CNS [138]. However, this is rather an advantage when the aim is to target the periphery. Third, chronic exposure to high concentrations of exogenous BDNF may induce TrkB receptor down-regulation, removal from the cell surface, and/or desensitisation, paradoxically reducing responsiveness to the neurotrophic signal over time. This homeostatic regulation of the receptor represents a critical limitation regarding strategies based on persistent CNS delivery [7], but approaches to potentially counteract this are possible, such as the combined overexpression of both BDNF and TrkB [139,140]. Collectively, findings suggest that a primary translational barrier to BDNF-based therapies arises not only from insufficient BDNF availability but, more importantly, from the requirement for precise spatiotemporal control over BDNF-TrkB signalling. Indeed, across neuropathologies, BDNF dysfunction most often reflects impaired trafficking, altered receptor processing or an imbalance between TrkB.FL and its truncated form, rather than simply a reduction in BDNF expression.

**Table 1:** Summary of therapeutic approaches that harness BDNF/TrkB to treat neuropathologies

Therapy	Mechanism	Advantages	Limitations	Clinical status
AAV gene therapy	Viral vector-mediated BDNF delivery	Long-term expression, targeted delivery, BBB penetration	Invasive, immune response, may lack regulation, irreversible	Phase I (AD NCT05040217) and preclinical (CMT)
TrkB agonist antibodies	Selective TrkB activation	Target specificity, no pro-apoptotic effects, engineerable	Poor CNS penetration, high cost, immunogenicity	Preclinical
Small molecule TrkB agonists	TrkB binding/activation	Oral bioavailability, BBB penetration, low cost	Rapid metabolism, off-targets effects	Preclinical (7,8-DHF, R13, LM22B-10)
Cell-based therapies	Transplanted BDNF-secreting cells	Local sustained delivery	Poor engraftment, immune rejection, invasive	Preclinical
Intranasal delivery	Brain delivery through the nose	Non-invasive, BBB penetration, rapid onset	Limited/variable delivery	Preclinical
Physical exercise	Activity-dependent up-regulation of endogenous BDNF	Non-pharmacological, pleiotropic effects	Confounded by BDNF-independent effects, modest BDNF increase	Phase III (ongoing PD trials)

AAV, adeno-associated virus; AD, Alzheimer’s disease; BBB, blood-brain barrier; CMT, Charcot–Marie–Tooth disease; CNS, central nervous system; PD, Parkinson’s disease; TrkB, tropomyosin receptor kinase B.

Therefore, directly administered CNS gene therapy (as in the ongoing AAV2-BDNF AD trial, NCT05040217), small molecule TrkB agonists with improved penetration, or interventions that enhance endogenous BDNF production have gained traction. However, this entails prioritising invasive injections over systemic delivery, increasing procedural risk (e.g., intrathecal injections versus subcutaneous), and reducing suitability for chronic treatment [141]. Indeed, vector-mediated gene therapy and cell transplantation strategies raise concerns regarding immunogenicity, the potential for off-targets effects in healthy tissues, and uncontrolled transgene expression [142]. Strategies to counteract these issues exist (e.g., immunosuppression, combining engineered capsids with tissue-specific promoters, and drug-elicitable AAVs), but they represent additional hurdles to translation. Importantly, patient heterogeneity, including genetic background, comorbidities, disease stage, and BDNF/TrkB polymorphisms, may also influence treatment responsiveness. Indeed, Val66Met reduces activity-dependent BDNF secretion and alters TrkB-mediated plasticity [143,144]. This single nucleotide polymorphism may thus influence both disease susceptibility and responsiveness to BDNF-enhancing interventions [145,146], yet is not routinely stratified in clinical trials.

Another tested strategy is the use of exercise to enhance BDNF expression and signalling. While this method is non-invasive and broadly beneficial, it has such pleiotropic impacts (e.g., on inflammation, vascularity, and metabolism), making the contribution of BDNF almost impossible to parse [105,106,110,116]. Moreover, the conflicting results regarding the use of BDNF as a biomarker highlight the existence of methodological and biological sources of variability. A notable issue is the biofluid selected for BDNF measurement, as serum contains BDNF released from platelets during coagulation. As pointed out in the literature, this represents an important species-specific issue since mouse platelets do not contain large stores of BDNF compared with rats or humans [7]. Additionally, BDNF isoform ratios and genetic polymorphisms should be integrated into diagnostic profiling and trial design to best evaluate whether BDNF is indeed an efficient biomarker.

Finally, the lack of information regarding long-term safety and efficacy from human trials underscores our incomplete understanding of BDNF signalling modulation in neurological diseases where long-term treatments are likely required.

### Perspectives

Despite the historical failures of systemic recombinant BDNF delivery, recent advances in drug development, gene therapy, and our understanding of BDNF biology have paved the way for strategies designed to overcome previous obstacles. As mentioned, the AAV-mediated gene therapy being tested in the ongoing Phase I clinical trial in AD patients, which employs injection of AAV2-BDNF directly into the entorhinal cortex, addresses the fundamental pharmacokinetic limitations [58,147]. It could potentially achieve sustained, localised BDNF expression at the site of pathology, while avoiding systemic exposure and potential off-target effects. Though critical questions remain regarding optimal vector capsid and potential immune responses, as well as the long-term consequences of BDNF overexpression in the absence of activity-dependent regulation. A second promising strategy involves the development of TrkB-specific agonist antibodies designed to selectively activate TrkB. However, antibody-based therapeutics face challenges including limited CNS penetration, high manufacturing costs, potential immunogenicity, and the question of whether prolonged TrkB activation without activity-dependent regulation might lead to receptor desensitisation or maladaptive signalling [148–150]. The third major approach involves small molecule TrkB agonists, mostly 7,8-DHF and its optimised derivatives. While 7,8-DHF has shown efficacy in numerous preclinical models across multiple neuropathologies, and offers the advantages of oral bioavailability, BBB penetration, and low cost, significant controversy surrounds its mechanism of action and specificity [151,152]. Initial reports suggested direct TrkB binding and activation, but other studies have questioned whether 7,8-DHF achieves sufficient brain concentrations to directly activate TrkB, leaving the mechanism of action of TrkB agonists unresolved [153,154].

It has been shown that the therapeutic window for BDNF signalling varies across diseases and stages of pathology, with beneficial effects depending on injury phase, neuronal subtype and circuit activity. Therefore, each of these strategies must consider appropriate localisation, activity-dependence and receptor composition to effectively restore the physiological patterns of BDNF signalling, alongside timing, dosing, and patient selection according to symptom onset, disease and stage of pathology. Importantly, genetic stratification of the Val66Met genotype should be considered, offering comprehensive understanding of patient heterogeneity and disease mechanisms to finally achieve BDNF’s therapeutic promise.

## Conclusion

BDNF has emerged as a crucial factor in a range of neurological disorders. Its roles in neurogenesis, synaptic plasticity, and neuroprotection highlight the therapeutic potential of targeting BDNF-TrkB signalling to mitigate disease progression and improve clinical outcomes. However, the complexities of BDNF regulation and signalling, and its effects across different conditions require further investigation, as do new strategies to overcome obstacles hindering its therapeutic potential [155]. Nevertheless, recent evidence continues to support the hypothesis that harnessing BDNF, either through pharmacological means or lifestyle interventions, such as physical exercise, holds promise as a therapeutic strategy for treating neuropathologies of both the CNS and PNS.

## Key open questions

- Which cells, circuits, and disease stages require intervention to maximise benefit of BDNF/TrkB therapeutics?
- Does chronic enhancement of BDNF signalling promote receptor desensitisation, hyperexcitability, and/or pain?
- How should genetic background, disease stage, and baseline BDNF signalling guide patient stratification and trial design?

## Perspectives

- BDNF is an essential neurotrophic factor that drives development, function, and survival of neurons within both the central and peripheral nervous systems.
- Impairments in BDNF expression, localisation, and/or signalling have been identified across neurological diseases, leading to the exploration of BDNF as a potential therapeutic target.
- Continued study of the production, processing, and activity of BDNF across the nervous system, both developmentally and with age, will clarify its function and lead to improved understanding of how it can be targeted to alleviate neuropathologies.

## Open Access

Open access for this article was enabled by the participation of University College London in an all-inclusive Read & Publish agreement with Portland Press and the Biochemical Society under an agreement with JISC.

## Abbreviations

AAV: adeno-associated virus
AD: Alzheimer's disease
ARS: aminoacyl-tRNA synthetase
Aβ: amyloid beta peptide
ALS: amyotrophic lateral sclerosis
BBB: blood–brain barrier
BDNF: brain-derived neurotrophic factor
CNS: central nervous system
CREB: cAMP response element-binding protein
CSF: cerebrospinal fluid
CMT: Charcot–Marie–Tooth disease
DPN: diabetic peripheral neuropathy
ER: endoplasmic reticulum
GlyRS: glycyl-tRNA synthetase
HAP1: huntingtin-associated protein-1
HD: Huntington's disease
HTT: huntingtin
mBDNF: mature BDNF
MN: motor neuron
mHTT: mutant huntingtin
NMJ: neuromuscular junction
p75^NTR^: p75 neurotrophin receptor
PD: Parkinson's disease
PNS: peripheral nervous system
SBMA: spinal-bulbar muscular atrophy
SMA: spinal muscular atrophy
TBI: traumatic brain injury
TrkB: tropomyosin receptor kinase B
7,8-DHF: 7,8-dihydroxyflavone
